# A Novel Adaptation Mechanism Underpinning Algal Colonization of a Nuclear Fuel Storage Pond

**DOI:** 10.1128/mBio.02395-17

**Published:** 2018-06-26

**Authors:** Victoria E. MeGraw, Ashley R. Brown, Christopher Boothman, Royston Goodacre, Katherine Morris, David Sigee, Lizzie Anderson, Jonathan R. Lloyd

**Affiliations:** a Research Centre for Radwaste Disposal, School of Earth and Environmental Sciences, The University of Manchester, Manchester, United Kingdom; b Williamson Research Centre for Molecular Environmental Science, School of Earth and Environmental Sciences, The University of Manchester, Manchester, United Kingdom; c Manchester Institute of Biotechnology, The University of Manchester, Manchester, United Kingdom; d Thorp Management Centre, Sellafield, Seascale, United Kingdom; CEH-Oxford

**Keywords:** *Haematococcus*, microbial ecology, nuclear waste, spent nuclear fuel

## Abstract

Geochemical analyses alongside molecular techniques were used to characterize the microbial ecology and biogeochemistry of an outdoor spent nuclear fuel storage pond at Sellafield, United Kingdom, that is susceptible to seasonal algal blooms that cause plant downtime. 18S rRNA gene profiling of the filtered biomass samples showed the increasing dominance of a species closely related to the alga Haematococcus pluvialis, alongside 16S rRNA genes affiliated with a diversity of freshwater bacteria, including *Proteobacteria* and *Cyanobacteria*. High retention of ^137^Cs and ^90^Sr on pond water filters coincided with high levels of microbial biomass in the pond, suggesting that microbial colonization may have an important control on radionuclide fate in the pond. To interpret the unexpected dominance of *Haematococcus* species during bloom events in this extreme environment, the physiological response of H. pluvialis to environmentally relevant ionizing radiation doses was assessed. Irradiated laboratory cultures produced significant quantities of the antioxidant astaxanthin, consistent with pigmentation observed in pond samples. Fourier transform infrared (FT-IR) spectroscopy suggested that radiation did not have a widespread impact on the metabolic fingerprint of H. pluvialis in laboratory experiments, despite the 80-Gy dose. This study suggests that the production of astaxanthin-rich encysted cells may be related to the preservation of the *Haematococcus* phenotype, potentially allowing it to survive oxidative stress arising from radiation doses associated with the spent nuclear fuel. The oligotrophic and radiologically extreme conditions in this environment do not prevent extensive colonization by microbial communities, which play a defining role in controlling the biogeochemical fate of major radioactive species present.

## IMPORTANCE

Spent nuclear fuel is stored underwater in large ponds prior to processing and disposal. Such environments are intensively radioactive but can be colonized by microorganisms. Colonization of such inhospitable radioactive ponds is surprising, and the survival mechanisms that microbes use is of fundamental interest. It is also important to study these unusual ecosystems, as microbes growing in the pond waters may accumulate radionuclides present in the waters (for bioremediation applications), while high cell loads can hamper management of the ponds due to poor visibility. In this study, an outdoor pond at the U.K. Sellafield facility was colonized by a seasonal bloom of microorganisms, able to accumulate high levels of ^137^Cs and ^90^Sr and dominated by the alga *Haematococcus*. This organism is not normally associated with deep water bodies, but it can adapt to radioactive environments via the production of the pigment astaxanthin, which protects the cells from radiation damage.

## INTRODUCTION

The generation of nuclear power and the resultant radioactive wastes, which have the potential to contaminate the environment with radionuclides, have led to intense public and academic interest in environmental radioactivity. This has most recently been highlighted by the events at the Fukushima-Daiichi nuclear power complex in Japan, where reports estimate that ~10^16^ Bq of radioactive cesium and ~10^14^ Bq of radioactive strontium were released accidentally into the environment following the Tōhoku earthquake and tsunami on 11 March 2011 (1). Undoubtedly, there will be contaminated equipment, fuel rods, and storage ponds that contain elevated concentrations of these and other radionuclides as a result of the accident. Thus, the development of safe management processes for radioactive wastes includes consideration of the large and complex global legacy materials and facilities which have resulted from more than 50 years of nuclear power generation and clearly affects any development of new nuclear power stations.

Microorganisms have been shown to survive and colonize a broad range of extreme habitats (2, 3), and there have been sporadic reports suggesting microbial colonization of radioactive environments at nuclear facilities (4–11). These include nuclear fuel waste disposal containers (12), contaminated soils, including the Department of Energy Hanford Site in Washington (4) and the Sellafield nuclear facility in the United Kingdom (13), water surrounding the damaged core reactor at the Three Mile Island Nuclear Power Plant (14, 15), the walls of the damaged number four reactor of the Chernobyl Nuclear Power Plant in Ukraine (16), nuclear reactor cooling pool waters (17, 18), and spent fuel ponds (5–8, 10, 19).

The fate of radionuclides in the environment is controlled mainly by the interaction between the background matrix of the radioactive material, the often complex chemistry of the radionuclides in question, and a broad range of biogeochemical factors associated with the extant microbial communities in the environment that has been contaminated. Indeed, in recent years, it has become clear that microorganisms exhibit significant control on radionuclide mobility in these environments via processes, including biosorption, bioaccumulation, biotransformation, biomineralization, and microbially enhanced chemisorption (20, 21). These processes must be studied in more detail if we are to understand and control biological processes occurring in nuclear fuel cycle facilities, understand their impact on the long-term fate of stored radioactive materials, and harness their potential for the cost-effective bioremediation of sediments and waters influenced by radionuclides (22–25). This is an extreme challenge, as nuclear facilities, including fuel storage ponds, are some of the most inhospitable facilities that environmental scientists have worked in, and are subject to extremely tight regulatory control. Thus, as a consequence of both safety and access issues, they remain largely uncharacterized.

This study focuses on an outdoor spent nuclear fuel storage pond at Sellafield, in Cumbria, United Kingdom where seasonal microbial productivity significantly impacts plant operations at the site. The pond contains a dynamic inventory of spent nuclear fuel and has been operating since the early 1960s. This fuel has typically been derived from a fleet of power-generating light water reactors and is ultimately destined for reprocessing within the on-site reprocessing facility, THORP (thermal oxide reprocessing plant). The pond is open to the atmosphere and has steel-lined concrete walls, and as part of the pond management strategy, the demineralized water volume of ~14,000 m^3^ is purged (replaced) at an average rate of ~400 m^3^ a day. Most of the fuel in the pond is zirconium clad and has typically been stored in specialist metallic fuel storage containers known as multielement bottles (MEBs), which ideally isolate the spent fuel from bulk exchange with the pond water. As well as MEB storage, it is noteworthy that some of the spent fuel has been held in open-topped “skips” (large metal-bodied containers, typically used for refuse) within the pond, and the zirconium-clad fuel in these skips will have exchanged with bulk water, with presumably a higher radionuclide release than from the fuels stored in MEBs.

The outdoor pond is subject to sodium and chloride incursions due to Sellafield’s close proximity to the coast, e.g., sea spray. Furthermore, the pond has a known history of seasonal generation of biomass typically referred to on-site as “algal blooms.” This has led to visibility issues and resultant plant downtime during the summer months, affecting the forward management of the spent nuclear fuel inventory. As would be expected in a facility where the spent fuel inventory is being moved according to site operations, there is large variability in the measured radiation dose at the site, with dose rates at the MEB side panels up to ~14 Gy h^−1^. Gamma radiation dose rates from the MEBs are higher at the side panels of the containers, as there is less shielding compared to the top and bottom of the MEBs. Additionally, due to both attenuation of the radioactivity by water and to the inverse square law, dose rates drop off rapidly with distance from the MEBs. In day-to-day pond operations, MEBs are moved through the water column causing the pond contents to be disturbed, and localized high radiation fluxes can occur. Thus, the system is dynamic in terms of the dose rate that biota receive, and it is clear that any biological materials that are entrained within the pond circulation will need to withstand dose rates from ambient to extreme.

In addition to the radiation dose from the MEBs, the ponds also contain radionuclides from the stored fuels. The radionuclide inventory is dominated by the high-yield fission products ^137^Cs and ^90^Sr, which have intermediate half-lives (30 years and 28.8 years, respectively), and are present in high quantities in the spent fuel stored within the pond (26). Thus, pond waters and any resultant solids where ^137^Cs and ^90^Sr are present at elevated levels will need to be managed for several hundred years for radioactive decay to occur.

Understanding the microbial ecology and biogeochemistry of the Sellafield spent fuel storage ponds is an important first step required for minimizing plant downtime due to reduced visibility and will also inform decommissioning activities for fuel storage ponds worldwide. In addition, the evolving nuclear accident at Fukushima-Daiichi has generated a renewed interest in the management of radioactivity from accidental releases. It is clear that as a result of the series of accidents at the site, significant quantities of highly active effluents containing ^137^Cs and ^90^Sr have been generated and will continue to be held in storage during the ongoing efforts to stabilize the site (27). Indeed, there are likely to be parallels between extreme accident scenarios and controlled spent nuclear fuel pond environments that may assist in the longer-term management of both planned and accidental nuclear releases. The aim of this study was to characterize, in detail, the unique microbial ecology of a spent fuel pond throughout a microbial bloom and to investigate the impact of this increase in microbial biomass on the cycling of key radionuclides in the pond.

## RESULTS

Water samples were collected from Sellafield’s outdoor storage pond approximately every 7 days between May 2010 and October 2010 (*n* = 16). The sampling campaign was aimed at characterizing changes in microbial ecology, biogeochemistry, and radiochemistry during a microbial bloom event affecting plant operations. Identification of key prokaryotic and eukaryotic species and their contribution to the bacterial/algal bloom and water chemistry was undertaken to further understand the radionuclide-microorganism-biosphere interactions in this extreme environment. Significant shifts in the microbial ecology of the ponds were noted over the 6-month sample period and included a photosynthetic bloom that occurred with changes in the inventory of the major radionuclides in the pond waters.

### Seasonal variations in physical and chemical conditions.

**FIG 1:**
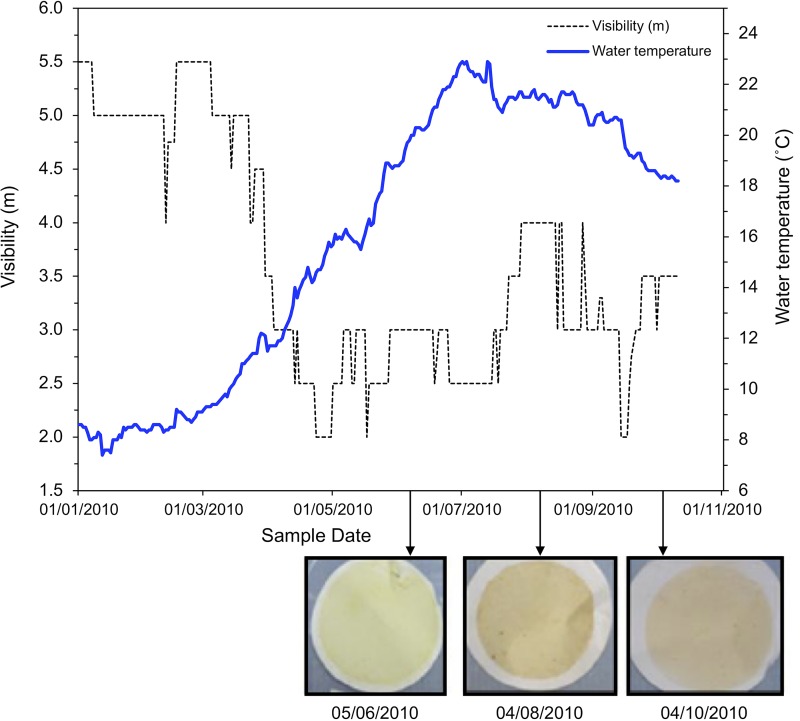
Sellafield spent nuclear fuel pond visibility (Secchi depth; the depth at which a black-and-white 20-cm “Secchi disk” becomes indistinguishable from the surrounding water) and pond surface water temperature. Inset images show filter papers used for molecular analyses. Dates in the figure are shown in the day/month/year format.

During the sampling campaign, pond water chemistry showed low overall variability (as expected, as the operational mode of the facility is designed to maintain highly controlled and constant conditions) with conductivity averaging 3.9 ± 0.6 µS cm^−1^ for all sample points and bulk pH changing between 6.4 and 8.0 (see Table S2 in the supplemental material). Additional data for the wider pond visibility and temperature were also made available by Sellafield Ltd. for the relevant sampling period, and there was a clear inverse relationship between temperature and visibility in the pond with the coldest water temperatures in January coinciding with a maximum visibility of ~5 m (Fig. 1). Pond water temperatures then increased from January to June as a result of increased solar radiation, with pond visibility at a minimum from May to July (<3 m). This drop in visibility was coincident with site microbial blooms hampering pond operations and affecting spent fuel management. Visibility in the pond then remained below 4 m for the remainder of the sampling campaign.

### Biomass and chlorophyll concentrations.

**FIG 2:**
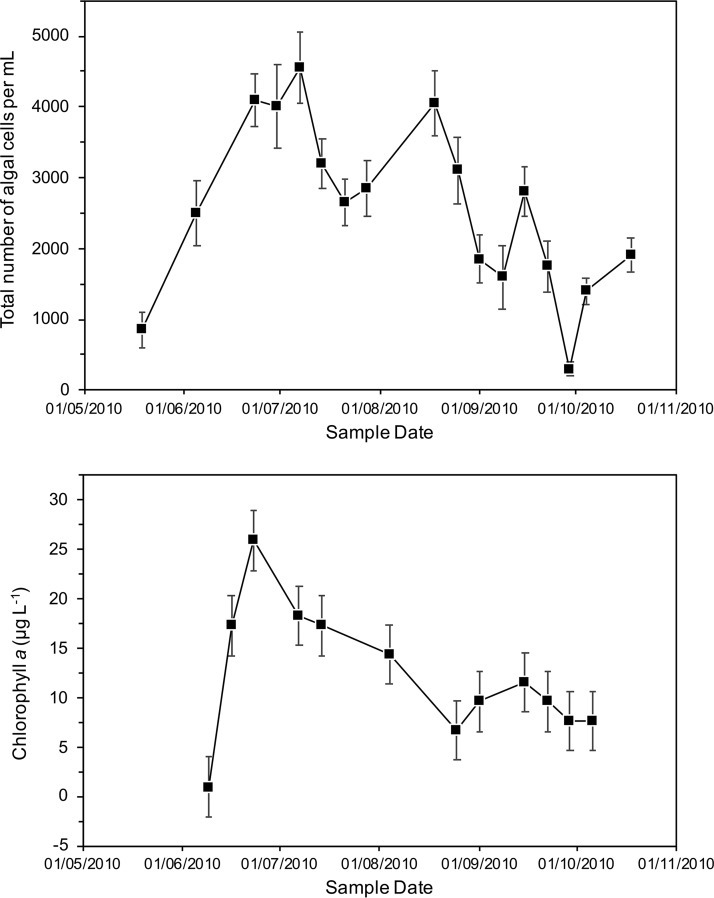
(Top) Total algal cell counts from May 2010 to October 2010. (Bottom) Chlorophyll *a* concentration from May 2010 to October 2010. Data points show the means of triplicate measurements, and error bars depict 1 standard deviation.

The chlorophyll *a* concentration, an indicator of trophic state (28), revealed that the system contained a large amount of biomass, typical of that of a biologically rich eutrophic water body during May 2010 to October 2010 and consistent with the visibility measurements. The water column visibility (Fig. 1) in addition to algal cell counts and chlorophyll *a* content (Fig. 2) indicated that the most significant biomass bloom occurred around July 2010. The filter from the prebloom June 2010 sample (when visibility was already poor) was pale yellow in color and had a relatively low chlorophyll *a* concentration (Fig. 1 and 2). In contrast, during the bloom period (mid-June to August), the filters were a red/orange color, and displayed consistently elevated chlorophyll *a* concentrations (Fig. 2). Additionally, the summer bloom also coincided with significantly higher concentrations of phosphate, nitrate, sulfate, calcium, and suspended solids in the pond (Table S2; data supplied by Sellafield Ltd.).

Microscopic algal cell counts (Fig. 2) showed that the number of cells over the sampling period ranged from 850 ± 244 cells per ml (on 19 May 2010) to a maximum of 4,550 ± 495 cells per ml (on 7 July 2010). The peak number of algal cells detected coincided with the time of the high pond turbidity and increased chlorophyll *a* concentrations in late June to early July. However, a slight fall in chlorophyll *a* concentrations through August, which does not directly correlate with high cell counts, may suggest a shift to production of red pigments that coincides with the filter color. There was then a general decline in algal cell counts through late summer and autumn to values approaching those at the start of the sampling in May 2010.

### Pond water radiochemistry.

For the radionuclide inventory, there was a general trend upwards in concentrations of both ^90^Sr and ^137^Cs in unfiltered waters over the sampling campaign. In unfiltered waters, the ^90^Sr concentration increased from 2.66 ± 0.07 Bq ml^−1^ in May 2010 to a maximum concentration of 5.94 ± 0.15 Bq ml^−1^ in late September 2010. The ^137^Cs concentration in the unfiltered water samples also increased gradually from 1.22 ± 0.03 Bq ml^−1^ in early June 2010 to a maximum concentration of 2.81 ± 0.07 Bq ml^−1^ in late September 2010. These increases in ^90^Sr and ^137^Cs concentrations throughout sampling are apparently unrelated to the broad peak in bulk biomass in late June to early July (Fig. 2).

**FIG 3:**
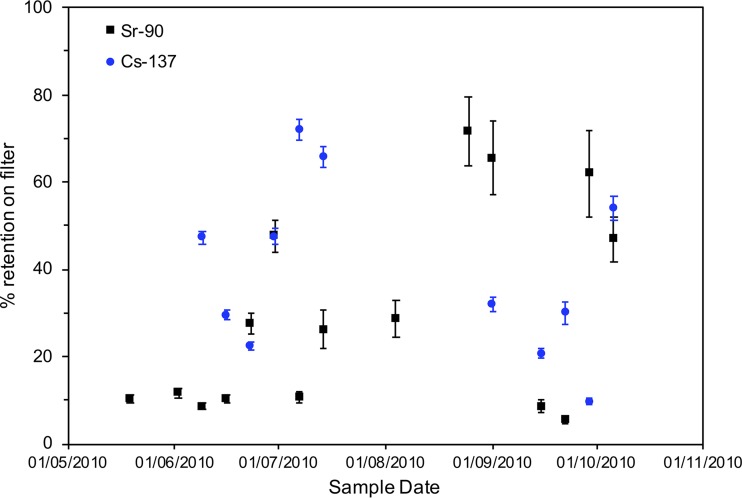
^137^Cs and ^90^Sr percentage retention on filter papers from May 2010 to October 2010. Data points show the means of triplicate measurements, and error bars depict 1 standard deviation.

The influence of biomass on radionuclide behavior in the system was further examined by considering radionuclide retention on the filtered biomass (Fig. 3). For ^90^Sr, the retention on the filters was highly variable throughout the year with between 5.6% ± 0.9% and 71.6% ± 7.9% retention observed at different time points. Interestingly, the three events with maximum retention occurred in late summer (25 August 2010, 1 September 2010, and 29 September 2010) with an average of 66.3% ± 4.9% of the ^90^Sr retained with the biomass at these time points. The remaining samples showed significantly lower retention with an average of 22.9% ± 15.1% associated with biomass at other time points. For ^137^Cs, again retention was variable, though a clear maximum of 72.1% ± 2.3% retention occurred during the peak in biomass observed in early July, followed by a general downward trend through the summer period with a final high retention observed on 6 October 2010 (Fig. 3).

### Microbial ecology and phylogenetic diversity of the pond.

**FIG 4:**
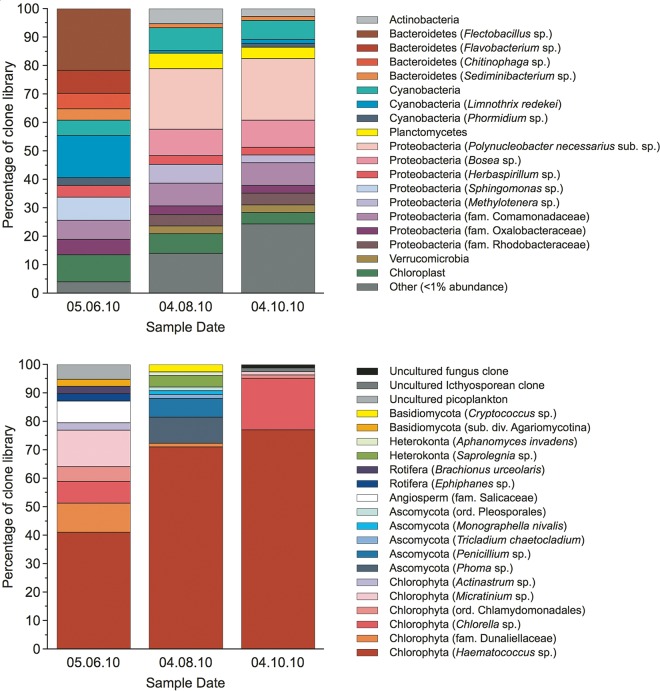
Phylogenetic affiliations (phyla, with closest distinguishable phylogenetic ranks in parentheses) of organisms detected in Sellafield pond samples using PCR with broad-specificity primers for prokaryotic 16S rRNA genes (top) and eukaryotic 18S rRNA genes (bottom). fam, family; sub. div. subdivision.

PCR amplifications using broad-specificity prokaryotic and eukaryotic primers produced a series of clone libraries showing a surprisingly rich microbial ecology, consisting of aquatic freshwater bacteria, freshwater algae, plankton, and to a lesser extent fungi and presumably reflecting the uncovered nature of the pond (Fig. 4 and Fig. S1 [Phyla] in the supplemental material). The neutral pH of the pond with associated marine salt incursions, ingress of windblown debris from the coast, and nutrient additions from sources, including guano and rainfall, undoubtedly play an important role in the development of such a complex ecosystem. Indeed, while there is no bulk exchange of seawater with the purged pond water, it is possible that there may be minor introduction of microbial biomass and associated DNA from the atmospheric sources mentioned above. However, the high pond biomass, evidenced through several bloom events with significant chlorophyll concentrations, suggests that the microbial community detected is a result of high net productivity of the pond community that developed *in situ*.

10.1128/mBio.02395-17.1 FIG S1  Phylogenetic affiliations (phyla) of organisms detected in Sellafield pond samples using PCR with broad-specificity primers for prokaryotic 16S rRNA genes (top) and eukaryotic 18S rRNA genes (bottom). Download FIG S1, TIF file, 1.9 MB. Copyright © 2018 MeGraw et al. 2018 MeGraw et al. This content is distributed under the terms of the Creative Commons Attribution 4.0 International license.

Of particular note was the unexpected dominance within the eukaryotic clone libraries of a close relative of Haematococcus pluvialis, a freshwater species of green alga, Chlorophyta (29), with a maximum dominance of the clone library occurring in October (~77%) (Fig. 4). Pyrosequencing technologies became available after the initial clone libraries had been generated in this study and were used to confirm our initial results from Sanger sequencing. The higher-throughput pyrosequencing data generated were in agreement with those of the clone libraries, confirming the dominance of a close H. pluvialis relative (H. pluvialis strain SAG 34-1b; 99.5% match [427/429] [30]) alongside a diverse prokaryotic community (Fig. S2).

10.1128/mBio.02395-17.2 FIG S2  Phylogenetic affiliations of organisms in Sellafield pond samples determined using pyrosequencing following PCR using broad-specificity primers for prokaryotic 16S rRNA genes (top) and eukaryotic 18S rRNA genes (bottom). A Haematococcus pluvialis strain (SAG 34-1b) was the closest known relative for 81.9%, 89.5%, and 88.6% of the eukaryotic community on each of the sampling dates, respectively (99.5% match in each case; 427/429) (D. Hepperle et al., J Mol Evol 47:420–430, 1998). PCR for 16S rRNA gene amplicon pyrosequencing was performed using tagged fusion bacterial primers 27F (D. J. Lane, p. 115–176, *in* E. Stackebrandt and M. Goodfellow, ed., *Nucleic Acid Techniques in Bacterial Systematics*, 1991) and 907R (G. Muyzer, A. Teske, C. O. Wirsen, and H. W. Jannasch, Arch Microbiol 164:165–172, 1995), targeting the V1-V5 hypervariable regions of the bacterial 16S rRNA gene. Primers were synthesized by IDTdna (Integrated DNA Technologies, BVBA, Leuven, Belgium). The fusion forward primer (5′ CCA TCT CAT CCC TGC GTG TCT CCG ACT CAG NNNNNNNNNN AG AGT TTG ATC MTG GCT CAG 3′) contained the 454 Life Sciences “Lib-L Primer A,” a 4-base “key” sequence (TCAG), a unique 10-base barcode “MID” sequence for each sample, and bacterial primer 27F. The reverse fusion primer (5′ CCT ATC CCC TGT GTG CCT TGG CAG TCT CAG CCG TCA ATT CMT TTR AGT TT 3′) contained the 454 Life Sciences “Lib-L Primer B,” a 4-base “key” sequence (TCAG), and bacterial primer 907R. PCR for 18S amplicon pyrosequencing was performed using tagged fusion bacterial primers F566 and R1438 (K. Hadziavdic et al., PLoS One 9:e87624, 2014), targeting the V3-V7 hypervariable regions of the eukaryote 18S rRNA gene. Primers were synthesized by IDTdna (Integrated DNA Technologies, BVBA, Leuven, Belgium). The fusion forward primer (5′ CCA TCT CAT CCC TGC GTG TCT CCG ACT CAG NNNNNNNNNN CAG CAG CCG CGG TAA TTC C 3′) contained the 454 Life Sciences “Lib-L Primer A,” a 4 base “key” sequence (TCAG), a unique 10-base barcode “MID” sequence for each sample, and eukaryote primer F566. The reverse fusion primer (5′ CCT ATC CCC TGT GTG CCT TGG CAG TCT CAG CAT CAC AGA CCT GTT ATT GC 3′) contained the 454 Life Sciences “Lib-L Primer B," a 4-base “key” sequence (TCAG), and eukaryote primer R1438. The pyrosequencing run was performed at The University of Manchester sequencing facility, using a Roche 454 Life Sciences GS Junior system. The 454 pyrosequencing reads were analyzed using Qiime 1.8.0 release (J. G. Caporaso et al., Nat Methods 7:335–336, 2010), and denoising and chimera removal were performed in Qiime during operational taxonomic unit (OTU) picking (at 97% sequence similarity) with usearch (R. C. Edgar, Bioinformatics 26:2460–2461, 2010). Taxonomic classification of all reads was performed in Qiime using the Ribosomal Database Project (RDP) at 90% confidence threshold (J. R. Cole et al., Nucleic Acids Res 37:D141−D145, 2009), while the closest GenBank match for the OTUs that contained the highest number of reads (the representative sequence for each OTU was used) was identified by BLASTN nucleotide search. Download FIG S2, TIF file, 0.3 MB. Copyright © 2018 MeGraw et al. 2018 MeGraw et al. This content is distributed under the terms of the Creative Commons Attribution 4.0 International license.

Haematococcus  pluvialis is well-known for its high content of the red ketocarotenoid pigment and strong antioxidant astaxanthin (3,3′-dihydroxy-β,β-carotene-4,4′-dione) which is important in aquaculture and various pharmaceuticals and cosmetics (31, 32). Astaxanthin is normally present at low concentrations within the cell, but it is accumulated rapidly when environmental conditions become unfavorable for normal cell growth (33). Microscopic analysis of pond samples illustrated a change in the putative *Haematococcus* cell morphology from small (5- to 10-µm), mobile, green cells with two flagella in the June samples to larger (10- to 30-µm) vegetative cells with no flagella and clearly with cysts with a red pigment, putatively identified as astaxanthin (34), in the September and October samples (Fig. S3). Interestingly, this change in the putative *Haematococcus* cell morphology to the moderately encysted vegetative stage coincides with a peak in ^90^Sr and ^137^Cs levels in the unfiltered pond waters, and is thus possibly related to radiation stress.

10.1128/mBio.02395-17.3 FIG S3  *Haematococcus* cells from pond water samples taken between September and October. Download FIG S3, TIF file, 0.3 MB. Copyright © 2018 MeGraw et al. 2018 MeGraw et al. This content is distributed under the terms of the Creative Commons Attribution 4.0 International license.

### Viability and encystation of experimentally irradiated H. pluvialis.

To investigate whether the dominance of *Haematococcus* in the clone library, alongside morphological changes recorded in the samples, may be the result of tolerance to radiation stress, pure cultures of H. pluvialis were irradiated with daily acute doses of 16-Gy X-ray radiation to a total absorbed dose of 80 Gy. Due to the constraints associated with working on a regulated nuclear site, it was not feasible to isolate *Haematococcus* from pond samples for further experimental work, so a pure culture of a very close relative was obtained from the Culture Collection of Algae and Protozoa (Scottish Marine Institute, Oban, United Kingdom). The dose regime chosen was also representative of radiation fluxes delivered to organisms in a flushed spent nuclear fuel pond, such as at Sellafield, where dose rates are dynamic, ranging from ambient to ~14 Gy h^−1^ at MEB surfaces. However, localized high radiation fluxes can occur as the spent nuclear fuel is moved through the water column.

**FIG 5:**
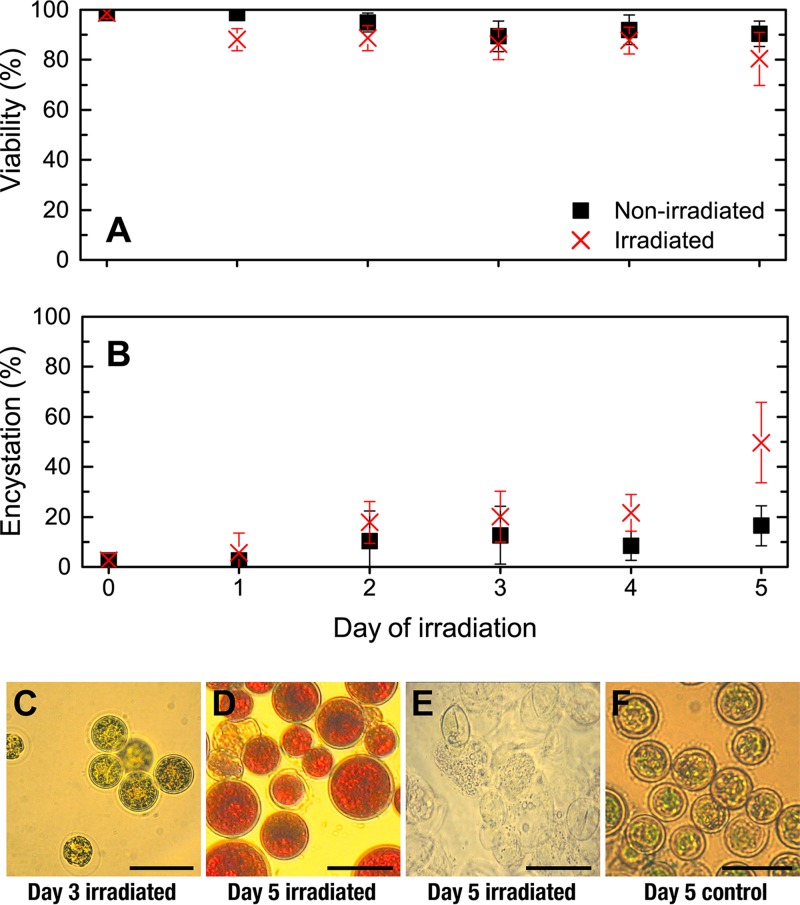
Effects of ionizing radiation on the viability and morphology of H. pluvialis cultures determined immediately after irradiation on each day. (A) Percentage of viable cells in irradiated and control cultures. Data points and error bars depict the means ± standard deviations of five replicate analyses from three biological replicates (*n* = 15). (B) Percentage of encysted cells in irradiated and control cultures. Data points and error bars depict the means ± standard deviations of five replicate analyses from three biological replicates (*n* = 15). (C) Irradiated cells after three consecutive days of irradiations showing palmelloid green cells. (D) Irradiated cells after five consecutive days of irradiations showing encysted cells and “ghost”/nonviable cells. (E) Irradiated cells showing “ghost” cells and cell debris after five consecutive days of irradiations. (F) Control culture after five consecutive days showing small palmelloid green cells. The images shown in panels D and E are both from the same sample, in which cell debris and “ghost” cells appeared to accumulate separately from encysted viable cells. Bars, 20 µm.

Viability remained similar for both control and irradiated cultures throughout the incubation period (Fig. 5A), suggesting that these doses did not have a significant impact on cellular physiology. However, by day 5, there was a slight drop in viability in irradiated cultures compared to controls. Although this was not significant (80% ± 11% and 90% ± 5%, respectively), it was accompanied by a noticeable increase in cell debris and “ghost” cells (dead cells that have lost their cytoplasmic contents and appear as a cell wall with clear interior) observed in irradiated cultures (Fig. 5E).

Analysis of cell morphology revealed an increase in encystation during the incubation period of the irradiated culture compared to the control culture (Fig. 5B and D). By day 5, the percentage of encysted cells in irradiated cultures was 50% ± 16% compared to 17% ± 8% in control cultures. Microscopy images also showed that by day 3, irradiated cultures were dominated by green immotile palmelloid (*Palmella*-like aggregates) cells (Fig. 5C), and by day 5, the majority of cells appeared larger, had thicker cell walls, and displayed increased astaxanthin accumulation, visible as red pigmentation (Fig. 5D). This was largely absent in nonirradiated control cultures (Fig. 5F).

### Analysis of H. pluvialis metabolic fingerprints by FT-IR spectroscopy.

**FIG 6:**
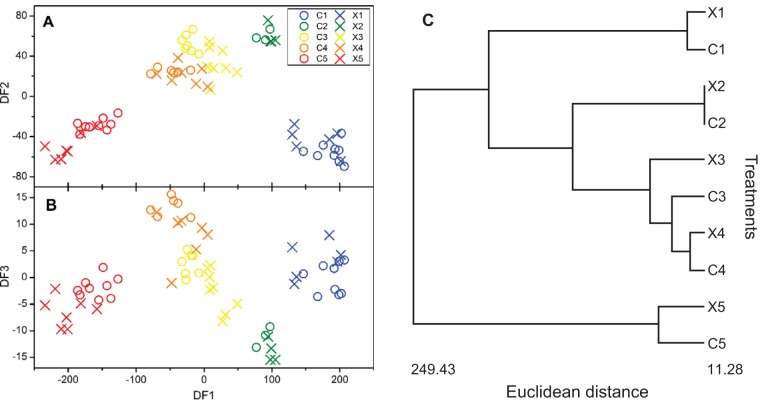
PC-DFA score plots of FT-IR spectra from all treatments. (A) Scores for discriminant function 1 (DF1) versus discriminant function 2 (DF2). (B) Scores of DF1 versus DF3. Twenty PCs were extracted from PCA and passed onto the DFA algorithm. (C) HCA dendrogram constructed from Euclidean distances between DF clusters in PC-DFA score plots of FT-IR spectra. Each treatment category represents the mean of each treatment class. C1 to C5 are control samples from day 1 (C1) to day 5 (C5), and X1 to X5 are irradiated samples from day 1 (X1) to day 5 (X5).

To quantify the impact of ionizing radiation on the metabolic fingerprints of H. pluvialis, infrared spectra were collected from both control and irradiated cultures immediately after each daily dose for 5 days (Fig. S4). The spectra from each treatment were qualitatively similar, making it difficult to discriminate between spectral features by visual inspection alone. Therefore, in order to detect the possible development of an irradiated phenotype, cluster analysis was performed using principal-component discriminant function analysis (PC-DFA). Figure 6A shows that the replicates of each treatment form tight clusters in the score plot of discriminant function 1 (DF1) versus DF2, suggesting that the metabolic fingerprints of cells from each treatment class were reproducible. In addition, the samples from both control and irradiated cultures taken on each treatment day cluster tightly together, such that day five control samples (C5), for example, appear most closely related to day five irradiated samples (X5) in the hierarchical cluster analysis (HCA) dendrogram (Fig. 6C). Likewise, there were no strong patterns of separation between clusters of control and treated samples in the score plot of DF1 versus DF3 (Fig. 6B). While there is some evidence of discrimination between respective irradiated and control clusters, Euclidean distances were not quantifiably related to dose (Fig. 6C).

10.1128/mBio.02395-17.4 FIG S4  Mean FT-IR spectra obtained from control (C) and irradiated (X) cultures throughout the 5-day experiment (day of treatment = 1, 2, 3, 4, and 5). The spectra are offset in the *y* axis for ease of visualization. Download FIG S4, TIF file, 0.1 MB. Copyright © 2018 MeGraw et al. 2018 MeGraw et al. This content is distributed under the terms of the Creative Commons Attribution 4.0 International license.

Collectively, these data indicate that the metabolic fingerprints of cells from both the control and irradiated cultures on each day are quantitatively very similar. Hence, it appears that total absorbed doses of up to 80 Gy had no discernible effect on the metabolic profiles detected by FT-IR spectroscopy, despite significant astaxanthin production as a result of irradiation (evidenced via cyst formation and pigmentation; Fig. 5).

## DISCUSSION

Despite the obvious challenges of working with materials from highly radioactive nuclear fuel storage facilities, it is becoming clear that microorganisms are uniquely able to colonize a range of nuclear sites worldwide  (5–8, 10). This study demonstrates the rich microbial ecology of an outdoor nuclear fuel pond environment, and in particular defines the species involved in microbial blooms at this site and explores their influence on the solubility of the radioactive inventory. Of particular interest is the dominance of a close relative to the freshwater chlorophyte Haematococcus pluvialis in the eukaryote clone libraries at the end of the summer bloom period when the levels of the fission products ^90^Sr and ^137^Cs in the pond water are at a maximum, and presumably when radiation fluxes within the biomass are also relatively high. Although this species is widely distributed in most parts of the world, it is typically associated with small ephemeral pools which are often subjected to high UV radiation fluxes (35), rather than the large, flushed body of water with its high radionuclide inventory studied here. The dominance of this species in the clone library throughout the algal bloom period, alongside morphological changes recorded in the samples, suggest that this organism may have an adaptive advantage which allows it to thrive in the outdoor spent fuel pond environment.

Experimental irradiation of pure cultures of H. pluvialis indicated that ionizing radiation had little impact on cell viability or the metabolic profile over the time frame tested. However, this coincided with the development of an astaxanthin-accumulating phenotype, which we suggest afforded protection against reactive oxygen species (ROS) generated by ionizing radiation (36, 37). Intriguingly, this could imply that this species may be able to withstand the highly radioactive conditions in areas of the outdoor pond as a result of its ability to produce astaxanthin, and thus thrive as a significant primary producer within the surprisingly complex microbial community in this unique environment. It is perhaps surprising that the development of this phenotype was not revealed by FT-IR spectroscopy, as this technique has been used previously to demonstrate molecular diversity within species (38) and to discriminate between different algal species (39, 40). It may be that the metabolic fingerprint of H. pluvialis is dominated by biomolecules such as proteins or lipids, which may mask any subtle changes to other biochemical components such as carotenoids. Indeed, the FT-IR spectrum of astaxanthin exhibits absorbance at IR regions similar to those of lipids and proteins (41).

While direct spectroscopic determination of astaxanthin in laboratory cultures was hindered by the difficulty in homogenizing cysts for astaxanthin extraction, light microscopy illustrated a greater percentage of astaxanthin-rich nonmotile encysted cells with increased radiation dose. Carotenoids, such as astaxanthin, protect the photosynthetic apparatus from light-mediated stress by quenching triplet state chlorophyll molecules, singlet oxygen molecules, and other ROS formed within the chloroplast via photooxidation (42). Astaxanthin production in H. pluvialis has been observed under a range of environmental and metabolic stressors, including high UV fluxes, which generate ROS that can damage DNA, proteins, and membranes (31, 32). Hence, the production of this molecule in irradiated cultures is likely to quench and scavenge ROS generated by ionizing radiation within cultures of H. pluvialis and is likely to have contributed to the survival and eventual dominance of this organism in the algal bloom noted in this nuclear fuel storage pond.

Finally, there are clearly multiple controls on the radionuclide flux in the dynamic pond environment and on the radionuclide retention mechanisms operating throughout the sampling period. Nonetheless, it is apparent that the microbial biomass present in the pond, dominated by the *Haematococcus* species, can have a significant impact on radionuclide partitioning within the pond environment. The controls on ^137^Cs retention, which peaked during the peak bloom period and ^90^Sr, which peaked at the end of the summer are different but are likely linked to photosynthetic activity and biocycling in this complex environment. The photosynthetic activity of the biomass is likely to affect the behavior of radionuclides in several ways. First, during photosynthesis, alkaline conditions up to pH ~10.5 can be generated at the localized cell/water interface, due to consumption of CO_2_ (43). Under these conditions, sorption of cations is likely to be enhanced, as localized pH values rise, resulting in the biomass surfaces becoming negatively charged and thus more attractive for Sr^2+^ and Cs^+^ sorption (44–48). Additionally, high pH favors biomineralization of carbonate phases, which may affect ^90^Sr behavior via incorporation into the biomineral matrix (44, 47, 49). Indeed, when we modeled pond water chemistries, open to the atmosphere and across a pH gradient between 7 and 10 (reflecting credible pH variations within the local biomass environment under photosynthetic conditions [28]), calcite (CaCO_3_) oversaturation was predicted at pH values greater than 9.2 (see Table S1 in the supplemental material). Importantly, Sr^2+^ is well-known to incorporate into carbonate mineral phases (47, 49–53), and thus it appears that in this system bioprecipitation of calcium carbonates due to pH elevation during photosynthesis may contribute to a localized effect where enhanced ^90^Sr retention may occur (54, 55). In addition, it is possible that biological uptake of these radionuclides may also have occurred (e.g., Cs^+^ substitution for K^+^ [56, 57] and Sr^2+^ substitution for Ca^2+^ [58]), with higher uptake and retention possibly resulting from the increased algal cell numbers during the bloom period.

10.1128/mBio.02395-17.5 TABLE S1  Calcite saturation in pond waters modeled over a range of pH. PHREEQC model parameters are reported in the article. Download TABLE S1, PDF file, 0.02 MB. Copyright © 2018 MeGraw et al. 2018 MeGraw et al. This content is distributed under the terms of the Creative Commons Attribution 4.0 International license.

In summary, the initial primary aim of this work was to gain fundamental insight into the microbial ecology of a nuclear fuel storage pond, identifying the key organisms that were able to colonize this highly radioactive environment and the cellular adaptation mechanisms at play. These are clearly key steps in understanding how to control microbial biomass production in this large facility, which complicates pond management. However, the bioaccumulation of key radionuclides by the robust microbial community studied here suggests that there could be an important role for such organisms in the sustainable bioremediation of highly radioactive waters in such facilities worldwide, and also in natural aquatic environments contaminated with radionuclides, including ^90^Sr and ^137^Cs.

## MATERIALS AND METHODS

### Safety.

Samples from a nuclear licensed site are subject to strict protocols during sampling and extensive monitoring procedures prior to shipping to any external facility. Samples containing radionuclides must be handled only by suitably qualified and experienced personnel in a properly equipped radiochemistry laboratory. The possession and use of radioactive materials are subject to statutory controls.

### Water sample collection, storage, and characterization.

For this study, a custom-made monitoring program was employed, with grab samples of water from the outdoor spent fuel storage pond collected on an approximately weekly basis from May 2010 to October 2010 (*n* = 16). Samples were collected from approximately 10 cm below the pond surface in sterile 500-ml bottles. Gross alpha and beta measurements were taken at Sellafield, and after appropriate checks, samples were then transported to The University of Manchester within 2 days upon receipt. The samples were stored in a refrigerator at 4°C in the dark. The samples were then filtered (0.2-µm Supor 47-mm filter; VWR, Chicago, IL, USA), typically within 24 h of receipt, and the filtrate and filters were stored immediately at 4°C in sterile containers prior to molecular ecology and radiometric and chemical characterization. Samples were analyzed as received (“unfiltered”), and a select series of biomass samples were also analyzed after filtration for molecular ecology and radiochemical and chemical characterization.

### Extraction, amplification, and sequencing of 16S and 18S rRNA genes.

DNA was extracted from filtered biomass using a PowerSoil DNA isolation kit (Mo Bio Laboratories, Inc., Solana Beach, CA, USA). Extracted 16S and 18S rRNA genes (typically 1,500 bp in length) were selectively amplified by PCR, using oligodeoxynucleotide primers. Purified DNA (2 µl) and 1 µl of 25 µM primer stocks were added to the reaction mix to a final volume of 50 µl. Primers used for bacterial 16S rRNA gene amplification were the broad-specificity 8F forward primer and the reverse primer 1492R (59). Primers used for the eukaryote 18S rRNA gene amplification were Euk F and Euk R (60). Samples were amplified using an iCycler (Bio-Rad) thermal cycler. Here the PCR mixtures contained 5 µl PCR buffer, 5 µl of 25 mM MgCl solution, 1 µl of 10 mM deoxynucleoside triphosphate (dNTP) solution, 0.5 µl of 25 µM primer, and 0.5 µl Sigma *Taq* DNA polymerase, which was made up to a final volume of 50 µl with sterile water. Thermal cycling was performed as follows for the bacterial 8F and 1492R primers: 35 cycles of PCR, with one cycle consisting of initial denaturation at 94°C for 4 min, melting at 94°C for 30 s, annealing at 55°C for 30 s, and extension at 72°C for 1 min with the final extension step of 72°C for 5 min (59). For eukaryotic 18S rRNA gene amplification, the following temperature cycle was used: 30 cycles with 1 cycle consisting of denaturation at 95°C for 1.5 min, annealing at 55°C for 1.5 min, and extension at 72°C for 1.5 min (60). The purity of the amplified PCR products was determined by electrophoresis in Tris-acetic acid-EDTA (TAE) gel 1% (wt/vol). DNA was stained with ethidium bromide and viewed under short-wavelength UV light using a Bio-Rad Geldoc 2000 system (Bio-Rad, Hemel Hempstead, Herts, UK).

PCR products were then purified further using a QIAquick PCR purification kit (Qiagen) and ligated directly into the cloning vector pCR 2.1 (Invitrogen) before transformation into competent Escherichia coli cells. White transformants that grew on LB agar containing ampicillin (100 mg ml^−1^) and 40 ml of 40 mg ml^**−**1^ of 5-bromo-4-chloro-3-indolyl-β-d-galactopyranoside (X-Gal) were screened for an insert using PCR (61). Primers were complementary to the flanking regions of the PCR insertion site of the pCR 2.1 cloning vector (ST1F and ST1R primers). The gene products of the clones were treated with shrimp alkaline phosphatase (Promega, UK) and exonuclease I (New England Biolabs, UK) and sequenced directly using an ABI Prism BigDye terminator cycle sequencing kit (v3.1) (Applied Biosystems, UK) following the manufacturer’s instructions. Sequences were obtained using the reverse PCR primer 1492R for prokaryote sequences and the reverse Euk R primer for eukaryote sequences. DNA sequences were determined with a Life Technologies 3730XL sequencer at the Genome Analysis Centre (John Innes Genome Laboratory, Norwich, United Kingdom). Sequences (typically 500 bp in length) were analyzed against the NCBI (United States) database using BLAST program packages and matched to known 16S and 18S rRNA gene sequences. Gene sequences were aligned using ClustalX software package and corrected manually.

### Radionuclide analysis.

To determine ^137^Cs activity and retention on biomass, analysis of unfiltered and filtered pond waters was undertaken using gamma ray spectroscopy on samples in a standard geometry. The detector was calibrated using a certified standard solution of ^137^Cs, and analyses were performed using an Ortec High Purity germanium detector. For ^90^Sr analysis, Cerenkov counting (liquid scintillation counting of Cerenkov radiation) was performed (Packard Tri-Carb model 1900). Again, samples were calibrated against a certified standard solution of ^90^Sr and in a standard volume to obtain the counting efficiency (62). In order to assess the error on these samples, triplicate analysis of a filtered water sample was undertaken, and the standard deviations of the measurements were calculated.

### PHREEQC analysis.

PHREEQC, version 2 (63), using the WATEQ4F.dat data set (64) was used to model the chemical speciation and saturation index of the representative pond water chemistry in a range of scenarios relevant to algal photosynthetic activity. The saturation index [SI = log(IAP/*K*_*T*_), where *K*_*T*_ is the solubility product constant and IAP is the corresponding observed activity product] provides a thermodynamic basis for indicating whether a mineral phase is over- or undersaturated, and in this case, it has been used to assess the potential for biomineralization in the ponds (64).

### Chlorophyll *a* analysis.

Chlorophyll *a* in the Sellafield pond samples was used as a proxy for total phototrophic biomass. A known volume of pond water was collected and filtered (0.2-µm Supor 47-mm filter; VWR, Chicago, IL, USA), stored at −20°C until analysis of samples (typically after 2 weeks), then extracted with 96% ethanol for 20 h at 5°C before centrifugation for 10 min at 2,000 × *g* (65). The UV absorbance of the supernatant at 665 nm (chlorophyll *a*) and 750 nm (correction for turbidity) was then determined on a Jenway 6700 UV-visible (UV-Vis) spectrophotometer.

### Algal counts.

Small subsamples from the spent nuclear fuel pond were preserved with Lugol’s iodine within 2 h of sample collection, affording a unique opportunity to analyze the algal biomass at the species level using microscopic counts (29). After full radiological safety assessment, enumeration was conducted on 1-ml samples of pond water, giving sufficiently large count numbers for statistical validity.

### Irradiation of Haematococcus pluvialis.

Unialgal cultures of H. pluvialis (strain H. pluvialis Flotow [1844] CCAP 34/7 1) were obtained from the Culture Collection of Algae and Protozoa (Scottish Marine Institute, Oban, UK). Axenic H. pluvialis cultures were grown phototrophically in presterilized Jaworski’s medium, according to the guidelines from the Culture Collection of Algae and Protozoa, Catalog of Strains 1988 (adapted for freshwater algae). Stock cultures were grown in 500-ml Erlenmeyer flasks, incubated at 24°C ± 1°C, and shaken at 100 rpm. The photon flux density (PFD) was maintained at 80 µmol m^−2^ s^−1^ with a 16-h:8-h light-dark cycle (supplied by cool fluorescent daylight lamps). For irradiation, three 20-ml biological replicates were prepared by inoculating fresh Jaworski’s medium with stationary-phase stock cultures which had been incubated for 10 days (optical density at 625 nm [OD_625_] of 0.17; approximately 7.2 × 10^4^ cells ml^−1^). These cultures were irradiated with 16 Gy X-radiation at ambient room temperature using a Faxitron CP-160 Cabinet X-radiator (160 kV; 6 mA; tungsten target). The dose rate was determined as 0.8 Gy min^−1^ using Fricke dosimetry (66, 67). A further three biological replicates formed the nonirradiated control cultures. These cultures were placed inside the irradiator to control temperature and were shielded by an appropriate thickness of lead. After irradiation, all cultures were incubated at 24°C ± 1°C and shaken at 100 rpm in a light incubator with a photon flux density of 80 µmol m^−2^ s^−1^ with a 16-h:8-h light-dark cycle. Irradiations, and subsequent analysis, were repeated each day for 5 days, giving a total absorbed dose of 80 Gy.

### Algal cell number and viability in irradiated laboratory cultures.

Cell concentration and viability were determined immediately after irradiation each day via five replicate cell counts using a Neubauer hemocytometer and a light microscope; Zeiss Axio Imager A1 (Carl Zeiss Microimaging GmbH, Germany). To determine cell viability, samples were stained using a fresh solution of Evans blue dye, an azo dye that penetrates nonviable cells only. Briefly, 200 mg of Evans blue (Sigma-Aldrich) in 10 ml phosphate-buffered saline (PBS) solution was added to the samples to give a final concentration of 0.1% (vol/vol) (40). The percentage of encysted cells was also determined by cell analysis under the light microscope. Encysted cells were identified by their larger size (>10 µm), lack of flagella (nonmotile), increased cell wall thickness and orange-red color (increased astaxanthin accumulation [34]). Additionally, cultures were photographed daily using a Zeiss microscope camera connector and a digital camera (Olympus B071).

### Analysis of metabolism by FT-IR spectroscopy.

The metabolic fingerprints of control and irradiated cells were recorded by Fourier transform infrared (FT-IR) spectroscopy. Aliquots were taken from each biological replicate immediately after irradiation each day. Samples were then centrifuged at 3,000 × *g* for 5 min; after centrifugation, the supernatant was removed, and the cell pellet was washed twice with sterile 0.9% NaCl solution prior to being centrifuged, flash frozen, and stored at −80°C. Upon analysis, samples were homogenized by freeze-thawing three times and then resuspended in sterile 0.9% NaCl solution to an OD_625_ of 1. A 96-well Si sample plate was washed thoroughly with 2-propanol and deionized water and allowed to dry at room temperature prior to use. Then, 20 µl of each algal sample was applied evenly in triplicate onto the plate at random locations (technical replicates) prior to drying at 55°C in an oven for 10 min. All FT-IR spectroscopic analyses were conducted using an Equinox 55 infrared spectrometer equipped with a high-throughput motorized microplate module, HTS-XT (Bruker Optics, Coventry, UK). A deuterated triglycine sulfate (DTGS) detector was employed for absorbance measurements of the samples to be acquired. Thus, three spectra from each biological replicate were collected over the wavenumber range of 4,000 to 600 cm^−1^ using the Opus software (Bruker Optics). Spectra were acquired at a resolution of 4 cm^−1^ with 64 spectra coadded and averaged to improve the signal-to-noise ratio. The collection time for each spectrum was approximately 1 min.

### Multivariate statistical analyses.

Prior to statistical analysis, FT-IR spectra were visually inspected and outlying spectra that deviated from the natural variability were removed from the data set. Features arising from CO_2_ at 2,400 to 2,275 cm^−1^ and below 700 cm^−1^ were removed from the spectra and replaced with a smoothed trend. An extended multiplicative scatter correction (EMSC) method was used to normalize spectra. This method was originally developed to reduce the effects of light scattering by particles and is particularly effective at removing noise and unavoidable intensity shifts from the spectra (68, 69). Prior to principal-component discriminant function analysis (PC-DFA) and hierarchical cluster analysis (HCA), spectra were scaled using an autoscaling process, such that for all spectra, the intensities at each wavenumber had a mean of zero and a standard deviation of one. Unsupervised principal-component analysis (PCA) was performed on the data to reduce data dimensionality (70). Principal components (PCs) were then extracted during this analysis, and discriminant function analysis (DFA) was applied to these PCs (i.e., PC-DFA). DFA is a supervised method, whereby discrimination between groups is based on *a priori* knowledge of experimental class structure. The algorithm acts to maximize between-group variance and minimize within-group variance. In this experiment, classes were assigned based on treatment and day of experiment. Discriminant function scores from PC-DFA were then used for HCA. The HCA algorithm uses the mean of the scores of each group to construct a dendrogram based on Euclidean distance between groups.

### Accession number(s).

DNA sequences from the Sellafield outdoor pond bacterial clone libraries have been submitted to GenBank under GenBank accession numbers KC009702 to KC009761.

10.1128/mBio.02395-17.6 TABLE S2  Concentrations of pond water constituents and pH during the sampling period. (Data supplied by Sellafield Ltd.) Download TABLE S2, PDF file, 0.04 MB. Copyright © 2018 MeGraw et al. 2018 MeGraw et al. This content is distributed under the terms of the Creative Commons Attribution 4.0 International license.

## References

[1] Steinhauser G, Brandl A, Johnson TE 2014 Comparison of the Chernobyl and Fukushima nuclear accidents: a review of the environmental impacts. Sci Total Environ 470–471:800–817. doi:10.1016/j.scitotenv.2013.10.029. 24189103

[2] Cavicchioli R 2002 Extremophiles and the search for extraterrestrial life. Astrobiology 2:281–292. doi:10.1089/153110702762027862. 12530238

[3] Rothschild LJ, Mancinelli RL 2001 Life in extreme environments. Nature 409:1092–1101. doi:10.1038/35059215. 11234023

[4] Fredrickson JK, Zachara JM, Balkwill DL, Kennedy D, Li SMW, Kostandarithes HM, Daly MJ, Romine MF, Brockman FJ 2004 Geomicrobiology of high-level nuclear waste-contaminated vadose sediments at the Hanford Site, Washington State. Appl Environ Microbiol 70:4230–4241. doi:10.1128/AEM.70.7.4230-4241.2004. 15240306 PMC444790

[5] Sarró MI, García AM, Moreno DA, Montero F 2007 Development and characterization of biofilms on stainless steel and titanium in spent nuclear fuel pools. J Ind Microbiol Biotechnol 34:433–441. doi:10.1007/s10295-007-0215-7. 17426994

[6] Sarró MI, Moreno DA, Chicote E, Lorenzo PI, García AM, Montero F 2003 Biofouling on austenitic stainless steels in spent nuclear fuel pools. Mater Corros 54:535–540. doi:10.1002/maco.200390117.

[7] Santo Domingo JW, Berry CJ, Summer M, Fliermans CB 1998 Microbiology of spent nuclear fuel storage basins. Curr Microbiol 37:387–394. doi:10.1007/s002849900398. 9806976

[8] Chicote E, García AM, Moreno DA, Sarró MI, Lorenzo PI, Montero F 2005 Isolation and identification of bacteria from spent nuclear fuel pools. J Ind Microbiol Biotechnol 32:155–162. doi:10.1007/s10295-005-0216-3. 15778866

[9] Lloyd JR, Renshaw JC 2005 Bioremediation of radioactive waste: radionuclide-microbe interactions in laboratory and field-scale studies. Curr Opin Biotechnol 16:254–260. doi:10.1016/j.copbio.2005.04.012. 15916892

[10] Chicote E, Moreno DA, Garcia AM, Sarro MI, Lorenzo PI, Montero F 2004 Biofouling on the walls of a spent nuclear fuel pool with radioactive ultrapure water. Biofouling 20:35–42. doi:10.1080/08927010410001662670. 15079891

[11] Nedelkova M, Merroun ML, Rossberg A, Hennig C, Selenska-Pobell S 2007 Microbacterium isolates from the vicinity of a radioactive waste depository and their interactions with uranium. FEMS Microbiol Ecol 59:694–705. doi:10.1111/j.1574-6941.2006.00261.x. 17381522

[12] Stroes-Gascoyne S, Pedersen K, Haveman SA, Dekeyser K, Arlinger J, Daumas S, Ekendahl S, Hallbeck L, Hamon CJ, Jahromi N, Delaney TL 1997 Occurrence and identification of microorganisms in compacted clay-based buffer material designed for use in a nuclear fuel waste disposal vault. Can J Microbiol 43:1133–1146. doi:10.1139/m97-162. 9476350

[13] McKenzie H, Armstrong-Pope N 2010 Groundwater annual report. Sellafield Ltd, Risley, Cheshire, United Kingdom.

[14] Booth W 1987 Postmortem on Three Mile Island: after 8 years and $1 billion, the cleanup is coming to an end. A mass of data has been produced but one nagging question remains: why wasn’t there core on the floor? Science 238:1342–1345. doi:10.1126/science.238.4832.1342. 17800559

[15] Romanovskaya VA, Rokitko PV, Mikheev AN, Gushcha NI, Malashenko YR, Chernaya NA 2002 The effect of γ-radiation and desiccation on the viability of the soil bacteria isolated from the alienated zone around the Chernobyl nuclear power plant. Microbiology 71:608–613. doi:10.1023/A:1020575223365. 12449639

[16] Mironenko NV, Alekhina IA, Zhdanova NN, Bulat SA 2000 Intraspecific variation in gamma radiation resistance and genomic structure in the filamentous fungus *Alternaria alternata*: a case study of strains inhabiting Chernobyl Reactor No. 4. Ecotoxicol Environ Saf 45:177–187. doi:10.1006/eesa.1999.1848. 10648134

[17] Mal’tsev VN, Saadavi A, Aiiad A, El’gaui O, Shlip M 1996 Microecology of nuclear reactor pool water. Radiats Biol Radioecol 36:52–57. (In Russian with English summary.) 8696485

[18] Rivasseau C, Farhi E, Compagnon E, de Gouvion Saint Cyr D, van Lis R, Falconet D, Kuntz M, Atteia A, Couté A 2016 Coccomyxa actinabiotis sp. nov. (Trebouxiophyceae, Chlorophyta), a new green microalga living in the spent fuel cooling pool of a nuclear reactor. J Phycol 52:689–703. doi:10.1111/jpy.12442. 27470701

[19] Galès G, Libert M-F, Sellier R, Cournac L, Chapon V, Heulin T 2004 Molecular hydrogen from water radiolysis as an energy source for bacterial growth in a basin containing irradiating waste. FEMS Microbiol Lett 240:155–162. doi:10.1016/j.femsle.2004.09.025. 15522503

[20] Macaskie LE, Lloyd JR 2002 Microbial interactions with radioactive wastes and potential applications, p 343–381. *In* Keith-Roach MJ, Livens FR (ed), Interactions of microorganisms with radionuclides. Elsevier, Oxford, United Kingdom.

[21] Lloyd JR, Macaskie LE 2000 Bioremediation of radionuclide-containing wastewaters, p 277–327. *In* Lovley DR (ed), Environmental microbe-metal interactions. ASM Press, Washington, DC.

[22] Gadd GM 2010 Metals, minerals and microbes: geomicrobiology and bioremediation. Microbiology 156:609–643. doi:10.1099/mic.0.037143-0. 20019082

[23] Geissler A, Selenska-Pobell S, Morris K, Burke IT, Livens FR, Lloyd JR 2010 The microbial ecology of land and water contaminated with radioactive waste: towards the development of bioremediation options for the nuclear industry, p 226–241. *In* Batty CL, Hallberg K, Jarvis PA (ed), The ecology of industrial pollution: restoration, remediation and preservation. Ecological reviews. Cambridge University Press, Cambridge, United Kingdom.

[24] Lloyd JR, Renshaw JC 2005 Microbial transformations of radionuclides: fundamental mechanisms and biogeochemical implications. Met Ions Biol Syst 44:205–240. 15971669

[25] Law GTW, Geissler A, Lloyd JR, Livens FR, Boothman C, Begg JDC, Denecke MA, Rothe J, Dardenne K, Burke IT, Charnock JM, Morris K 2010 Geomicrobiological redox cycling of the transuranic element neptunium. Environ Sci Technol 44:8924–8929. doi:10.1021/es101911v. 21047117

[26] Hunter J 2003 SCLS phase 1 conceptual model of contamination below ground at Sellafield. Nuclear Sciences and Technology Services report NSTS 4920. Nuclear Sciences and Technology Services, Las Vegas, NV.

[27] Ohta T, Mahara Y, Kubota T, Fukutani S, Fujiwara K, Takamiya K, Yoshinaga H, Mizuochi H, Igarashi T 2012 Prediction of groundwater contamination with ^137^Cs and ^131^I from the Fukushima nuclear accident in the Kanto District. J Environ Radioact 111:38–41. doi:10.1016/j.jenvrad.2011.11.017. 22209029

[28] Vollenweider RA, Kerekes JJ 1982 Background and summary results of the OECD co-operative programme on eutrophication. Organisation for Economic Co-operation and Development, Paris, France.

[29] Bellinger EG, Sigee DC 2010 Freshwater algae. John Wiley & Sons, Ltd, Chichester, United Kingdom.

[30] Hepperle D, Nozaki H, Hohenberger S, Huss VA, Morita E, Krienitz L 1998 Phylogenetic position of the Phacotaceae within the Chlamydophyceae as revealed by analysis of 18S rDNA and rbcL sequences. J Mol Evol 47:420–430. doi:10.1007/PL00006399. 9767687

[31] Borowitzka MA, Huisman JM, Osborn A 1991 Culture of the astaxanthin-producing green alga *Haematococcus pluvialis* 1. Effects of nutrients on growth and cell type. J Appl Phycol 3:295–304. doi:10.1007/BF02392882.

[32] Kobayashi M, Kakizono T, Nagai S 1991 Astaxanthin production by a green alga, *Haematococcus pluvialis* accompanied with morphological changes in acetate media. J Ferment Bioeng 71:335–339. doi:10.1016/0922-338X(91)90346-I.

[33] Boussiba S, Vonshak A 1991 Astaxanthin accumulation in the green alga *Haematococcus pluvialis*. Plant Cell Physiol 32:1077–1082. doi:10.1093/oxfordjournals.pcp.a078171.

[34] Buchheim MA, Sutherland DM, Buchheim JA, Wolf M 2013 The blood alga: phylogeny of Haematococcus (Chlorophyceae) inferred from ribosomal RNA gene sequence data. Eur J Phycol 48:318–329. doi:10.1080/09670262.2013.830344.

[35] Proctor VW 1957 Some controlling factors in the distribution of *Haematococcus pluvialis*. Ecology 38:457–462. doi:10.2307/1929890.

[36] Kobayashi M, Kakizono T, Nishio N, Nagai S, Kurimura Y, Tsuji Y 1997 Antioxidant role of astaxanthin in the green alga *Haematococcus pluvialis*. Appl Microbiol Biotechnol 48:351–356. 10.1007/s002530000416 11092631

[37] Wang S-B, Chen F, Sommerfeld M, Hu Q 2004 Proteomic analysis of molecular response to oxidative stress by the green alga *Haematococcus pluvialis* (Chlorophyceae). Planta 220:17–29. doi:10.1007/s00425-004-1323-5. 15258760

[38] Sigee DC, Dean A, Levado E, Tobin MJ 2002 Fourier-transform infrared spectroscopy of *Pediastrum duplex*: characterization of a micro-population isolated from a eutrophic lake. Eur J Phycol 37:19–26. doi:10.1017/S0967026201003444.

[39] Dean AP, Sigee DC 2006 Molecular heterogeneity in *Aphanizomenon flos-aquae* and *Anabaena flos-aquae* (Cyanophyta): a synchrotron-based Fourier-transform infrared study of lake micropopulations. Eur J Phycol 41:201–212. doi:10.1080/09670260600645907.

[40] Sigee DC, Selwyn A, Gallois P, Dean AP 2007 Patterns of cell death in freshwater colonial cyanobacteria during the late summer bloom. Phycologia 46:284–292. doi:10.2216/06-69.1.

[41] Chen X, Chen R, Guo Z, Li C, Li P 2007 The preparation and stability of the inclusion complex of astaxanthin with β-cyclodextrin. Food Chem 101:1580–1584. doi:10.1016/j.foodchem.2006.04.020. 26003346

[42] Young AJ 1991 The photoprotective role of carotenoids in higher plants. Physiol Plant 83:702–708.

[43] Lucas WJ, Smith FA 1973 The formation of alkaline and acid regions at the surface of *Chara corallina* cells. J Exp Bot 24:1–14. doi:10.1093/jxb/24.1.1.

[44] Thorpe CL, Morris K, Boothman C, Lloyd JR 2012 Alkaline Fe(III) reduction by a novel alkali-tolerant *Serratia* sp. isolated from surface sediments close to Sellafield nuclear facility, UK. FEMS Microbiol Lett 327:87–92. doi:10.1111/j.1574-6968.2011.02455.x. 22092936

[45] Chorover J, Choi S, Rotenberg P, Serne RJ, Rivera N, Strepka C, Thompson A, Mueller KT, O’Day PA 2008 Silicon control of strontium and cesium partitioning in hydroxide-weathered sediments. Geochim Cosmochim Acta 72:2024–2047. doi:10.1016/j.gca.2008.01.026.

[46] Roden EE, Leonardo MR, Ferris FG 2002 Immobilization of strontium during iron biomineralization coupled to dissimilatory hydrous ferric oxide reduction. Geochim Cosmochim Acta 66:2823–2839. doi:10.1016/S0016-7037(02)00878-5.

[47] Mitchell AC, Ferris FG 2005 The coprecipitation of Sr into calcite precipitates induced by bacterial ureolysis in artificial groundwater: temperature and kinetic dependence. Geochim Cosmochim Acta 69:4199–4210. doi:10.1016/j.gca.2005.03.014.

[48] Chen J-P 1997 Batch and continuous adsorption of strontium by plant root tissues. Bioresour Technol 60:185–189. doi:10.1016/S0960-8524(97)00021-7.

[49] Fujita Y, Redden GD, Ingram JC, Cortez MM, Ferris FG, Smith RW 2004 Strontium incorporation into calcite generated by bacterial ureolysis. Geochim Cosmochim Acta 68:3261–3270. doi:10.1016/j.gca.2003.12.018.

[50] Zachara JM, Cowan CE, Resch CT 1991 Sorption of divalent metals on calcite. Geochim Cosmochim Acta 55:1549–1562. doi:10.1016/0016-7037(91)90127-Q.

[51] Finch AA, Allison N, Sutton SR, Newville M 2003 Strontium in coral aragonite: 1. Characterization of Sr coordination by extended absorption X-ray fine structure. Geochim Cosmochim Acta 67:1197–1202. doi:10.1016/S0016-7037(02)01224-3.

[52] Greegor RB 1997 Strontianite in coral skeletal aragonite. Science 275:1452–1454. 9072808 10.1126/science.275.5305.1452

[53] Martin JB, Moore PJ 2008 Sr concentrations and isotope ratios as tracers of ground-water circulation in carbonate platforms: examples from San Salvador Island and Long Island, Bahamas. Chem Geol 249:52–65. doi:10.1016/j.chemgeo.2007.11.009.

[54] Stocks-Fischer S, Galinat JK, Bang SS 1999 Microbiological precipitation of Caco_3_. Soil Biol Biochem 31:1563–1571. doi:10.1016/S0038-0717(99)00082-6.

[55] Schultze-Lam S, Fortin D, Davis BS, Beveridge TJ 1996 Mineralization of bacterial surfaces. Chem Geol 132:171–181. doi:10.1016/S0009-2541(96)00053-8.

[56] Avery SV, Codd GA, Gadd GM 1992 Replacement of cellular potassium by caesium in Chlorella emersonii: differential sensitivity of photoautotrophic and chemoheterotrophic growth. J Gen Microbiol 138:69–76. doi:10.1099/00221287-138-1-69.

[57] Avery SV, Codd GA, Gadd GM 1993 Transport kinetics, cation inhibition and intracellular location of accumulated caesium in the green microalga Chlorella salina. J Gen Microbiol 139:827–834. doi:10.1099/00221287-139-4-827.

[58] Williams LG, Kevern NR 1964 Relative strontium and calcium uptake by green algae. Science 146:1488. 14208583 10.1126/science.146.3650.1488

[59] Eden PAE, Schmidt TM, Blakemore RP, Pace NR 1991 Phylogenetic analysis of *Aquaspirillum magnetotacticum* using polymerase chain reaction-amplified 16S rRNA-specific DNA. Int J Syst Bacteriol 41:324–325. doi:10.1099/00207713-41-2-324. 1854644

[60] DeLong EF 1992 Archaea in coastal marine environments. Proc Natl Acad Sci U S A 89:5685–5689. doi:10.1073/pnas.89.12.5685. 1608980 PMC49357

[61] Islam FS, Gault AG, Boothman C, Polya DA, Charnock JM, Chatterjee D, Lloyd JR 2004 Role of metal-reducing bacteria in arsenic release from Bengal Delta sediments. Nature 430:68–71. doi:10.1038/nature02638. 15229598

[62] Blackburn R, Al-Masri MS 1993 Radioassay of strontium-90 in the presence of calcium-45 and radiocaesium (^134^Cs and ^137^Cs). Appl Radiat Isot 44:683–686. doi:10.1016/0969-8043(93)90132-T.

[63] Parkhurst DL, Appelo CAJ 1999 User’s guide to PHREEQC (version 2)—a computer program for speciation, batch-reaction, one-dimensional transport and inverse geochemical calculations. Water-Resources Investigations Report 99-4259. US Geological Survey, Denver, CO.

[64] Ball JW, Nordstrom DK 1991 User's manual for WATEQ4F, with revised thermodynamic data base and text cases for calculating speciation of major, trace, and redox elements in natural waters. Open-File Report 91–183. U.S. Geological Survey, Denver CO.

[65] Jespersen A-M, Christoffersen K 1987 Measurements of chlorophyll―a from phytoplankton using ethanol as extraction solvent. Arch Hydrobiol 109:445–454.

[66] Fricke H, Morse S 1927 The chemical action of Roentgen rays on dilute ferrosulphate solutions as a measure of dose. Am J Roentgenol Radium Ther Nucl Med 18:430–432.

[67] Weiss J, Allen AO, Schwarz HO 1955 Use of the Fricke ferrous sulfate dosimeter for gamma ray doses in the range 4 to 40 kr, p 179–181. *In* Proceedings of the International Conference on the Peaceful Uses of Atomic Energy United Nations, New York, NY.

[68] Martens H, Stark E 1991 Extended multiplicative signal correction and spectral interference subtraction: new preprocessing methods for near infrared spectroscopy. J Pharm Biomed Anal 9:625–635. doi:10.1016/0731-7085(91)80188-F. 1790182

[69] Correa E, Sletta H, Ellis DI, Hoel S, Ertesvåg H, Ellingsen TE, Valla S, Goodacre R 2012 Rapid reagentless quantification of alginate biosynthesis in *Pseudomonas fluorescens* bacteria mutants using FT-IR spectroscopy coupled to multivariate partial least squares regression. Anal Bioanal Chem 403:2591–2599. doi:10.1007/s00216-012-6068-6. 22585056

[70] Goodacre R, Broadhurst D, Smilde AK, Kristal BS, Baker JD, Beger R, Bessant C, Connor S, Capuani G, Craig A, Ebbels T, Kell DB, Manetti C, Newton J, Paternostro G, Somorjai R, Sjöström M, Trygg J, Wulfert F 2007 Proposed minimum reporting standards for data analysis in metabolomics. Metabolomics 3:231–241. doi:10.1007/s11306-007-0081-3.

