# Supplementary figures and images for: A Novel Adaptation Mechanism Underpinning Algal Colonization of a Nuclear Fuel Storage Pond
Source: mBio. 2018 Jun 26;9(3):e02395-17. doi: 10.1128/mBio.02395-17 (PMC6020298; doi:10.1128/mBio.02395-17)

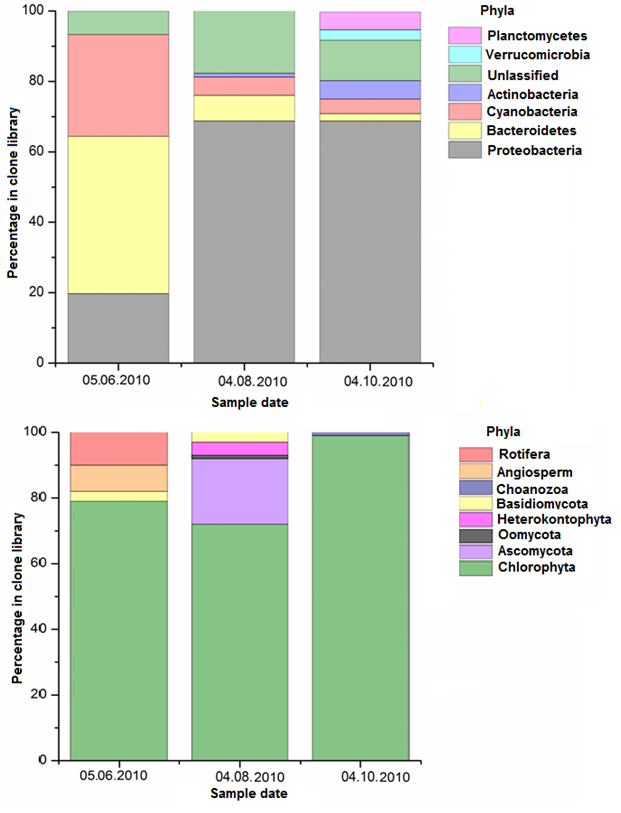

Supplement: FIG S1 [file mbo003183941sf1.tif]

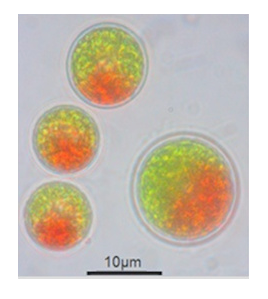

Supplement: FIG S3 [file mbo003183941sf3.tif]
