# Supplementary material for: A Novel Adaptation Mechanism Underpinning Algal Colonization of a Nuclear Fuel Storage Pond
Source: mBio. 2018 Jun 26;9(3):e02395-17. doi: 10.1128/mBio.02395-17 (PMC6020298; doi:10.1128/mBio.02395-17)
Supplement: TABLE S1 [file mbo003183941st1.pdf]

pH	Saturation Index $CaCO_3$
7	-4.55
8	-2.58
9	-0.78
9.15	-0.02
10	2.04
