# Supplementary material for: A Novel Adaptation Mechanism Underpinning Algal Colonization of a Nuclear Fuel Storage Pond
Source: mBio. 2018 Jun 26;9(3):e02395-17. doi: 10.1128/mBio.02395-17 (PMC6020298; doi:10.1128/mBio.02395-17)
Supplement: TABLE S2 [file mbo003183941st2.pdf]

**Table S2.** Concentrations of pond water constituents and pH during the sampling period. (Data supplied by Sellafield Ltd.)

| Date | pH | $Ca^{2+}$ mg / L | $Cl^{-}$ mg / L | $PO_4^{3-}$ mg / L | $NO_3^{-}$ mg / L | $SO_4^{2-}$ mg / L | Suspended solids mg / L |
|---|---|---|---|---|---|---|---|
| 05.04.2010 | 6.5 | 0.13 | 0.1 | 0 | 0 | 0.1 | 0.5 |
| 12.04.2010 | 6.6 | 0.15 | 0.2 | 0 | 0 | 0.1 | 1.7 |
| 19.04.2010 | 6.4 | 0.18 | 0.3 | 0 | 0 | 0.1 | 2.1 |
| 26.04.2010 | 6.9 | 0.17 | 0.3 | 0 | 0 | 0.2 | 2.4 |
| 03.05.2010 | 6.8 | 0.19 | 0.2 | 0 | 0 | 0.1 | 2.4 |
| 10.05.2010 | 8.0 | 0.22 | 0.4 | 0 | 0 | 0.2 | 3.6 |
| 17.05.2010 | 6.7 | 0.17 | 0.3 | 0 | 0 | 0.1 | 1.7 |
| 24.05.2010 | 7.0 | 0.22 | 0.3 | 0 | 0 | 0.2 | 2.2 |
| 31.05.2010 | 7.3 | 0.19 | 0.3 | 0 | 0 | 0.1 | 1.9 |
| 07.06.2010 | 6.7 | 0.53 | 0.5 | 0.6 | 0.4 | 0.5 | 25.7 |
| 14.06.2010 | 7.2 | 0.35 | 0.4 | 0 | 0 | 0.1 | 2.9 |
| 21.06.2010 | 6.6 | 0.21 | 0.3 | 0 | 0 | 0 | 5 |
| 28.06.2010 | 7.2 | 0.23 | 0.3 | 0 | 0 | 0.1 | 3 |
| 05.07.2010 | 7.4 | 0.23 | 0.4 | 0 | 0 | 0.1 | 2.5 |
| 12.07.2010 | 7.1 | 0.18 | 0.5 | 0 | 0 | 0.1 | 5.1 |
| 19.07.2010 | 7.4 | 0.19 | 0.5 | 0 | 0 | 0.1 | 2.1 |
| 26.07.2010 | 7.0 | 0.21 | 0.4 | 0 | 0 | 0 | 2.5 |
| 02.08.2010 | 7.2 | 0.26 | 0.5 | 0 | 0 | 0.1 | 2.3 |
| 09.08.2010 | 8.0 | 0.22 | 0.5 | 0 | 0 | 0.1 | 2.8 |
| 16.08.2010 | 6.9 | 0 | 0.3 | 0 | 0 | 0 | 2.1 |
| 23.08.2010 | 7.0 | 0.11 | 0.3 | 0 | 0 | 0 | 5.6 |
| 30.08.2010 | 6.8 | 0.3 | 0.7 | 0 | 0 | 0.1 | 7.1 |
| 06.09.2010 | 6.9 | - | 0.3 | 0 | 0.2 | 0 | 2.5 |
| 13.09.2010 | 7.2 | 0.33 | 0.2 | - | 0 | 0 | 2.4 |
| 20.09.2010 | 6.9 | 0.33 | 0.4 | 0 | 0 | 0 | 2 |
| 27.09.2010 | 7.8 | 0.82 | 2.1 | 0 | 1.2 | 1.6 | 2.2 |
